# Supplementary material for: Cationic Surfactant-Modified Tetraselmis sp. for the Removal of Organic Dyes from Aqueous Solution
Source: Molecules. 2023 Nov 29;28(23):7839. doi: 10.3390/molecules28237839 (PMC10708098; doi:10.3390/molecules28237839)
Supplement: Supplementary file 1 [file molecules-28-07839-s001.zip › molecules-2730271-supplementary.pdf]

Supplementary material for:

Cationic Surfactant-Modified *Tetraselmis* sp. for the Removal of Organic Dyes from Aqueous Solution

Buhani [1], Istikomah [1], Suharso [1], Sumadi [2], Sutarto [3], Huda M. Alghamdi [4] and Khalid Z. Elwakeel [4,5,*]

[1] Department of Chemistry, Faculty of Mathematic and Natural Sciences, University of Lampung, Jl. Soemantri Brojonegoro No. 1, Bandar Lampung 35145, Indonesia; buhani@fmipa.unila.ac.id (B.); isti9515@gmail.com (I.); suharso@fmipa.unila.ac.id (S.)

[2] Department of Electrical Engineering, Faculty of Engineering, University of Lampung, Jl. Soemantri Brojonegoro No. 1, Bandar Lampung 35145, Indonesia; sumadi.1973@eng.unila.ac.id

[3] Department of Medicine, Faculty of Medicine, University of Lampung, Bandar Lampung 35141, Indonesia

[4] Department of Chemistry, College of Science, University of Jeddah, Jeddah 80327, Saudi Arabia; halghamdi3@uj.edu.sa

[5] Environmental Chemistry Division, Environmental Science Department, Faculty of Science, Port Said University, Port Said 42522, Egypt

* Correspondence: kelwkeel@uj.edu.sa

Table S1: Chemical Properties and Characteristics of MO and MB

Generic name	Methyl orange	Methylene blue
Chemical formula	$C_{14}H_{14}N_3NaO_3S$	$C_{16}H_{18}ClN_3S$
Molecular weight ($g \cdot mol^{-1}$)	327.33	319.85
Type of dye	Anionic	Cationic
λ_{max} (nm)	465	664

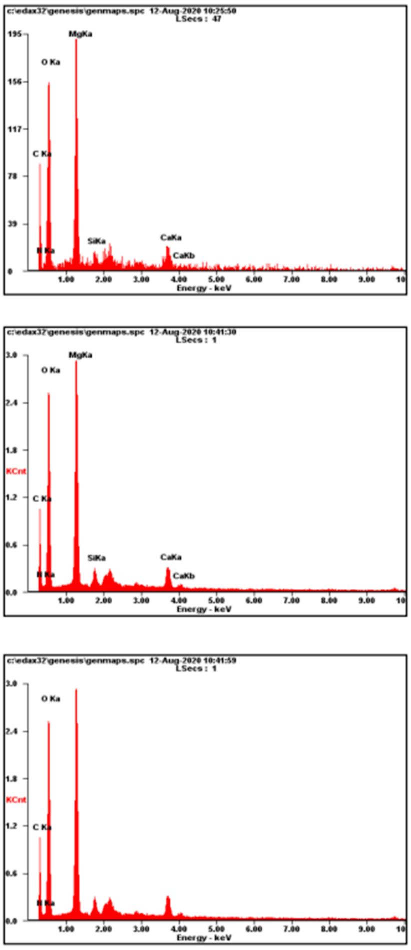

Element	Wt%	At%
CK	30.62	39.88
NK	01.82	02.03
OK	45.49	44.47
MgK	19.37	12.46
SiK	00.65	00.36
CaK	02.05	00.80
Matrix	Correction	ZAF

Element	Wt%	At%
CK	25.20	33.66
NK	04.76	05.45
OK	45.36	45.48
MgK	20.15	13.30
SiK	01.75	01.00
CaK	02.78	01.11
Matrix	Correction	ZAF

Element	Wt%	At%
CK	26.61	32.36
NK	04.89	05.10
OK	68.50	62.54
Matrix	Correction	ZAF

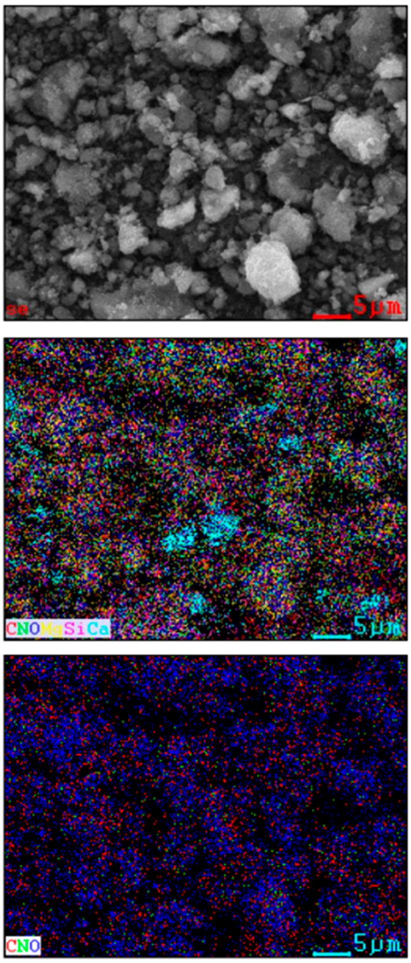

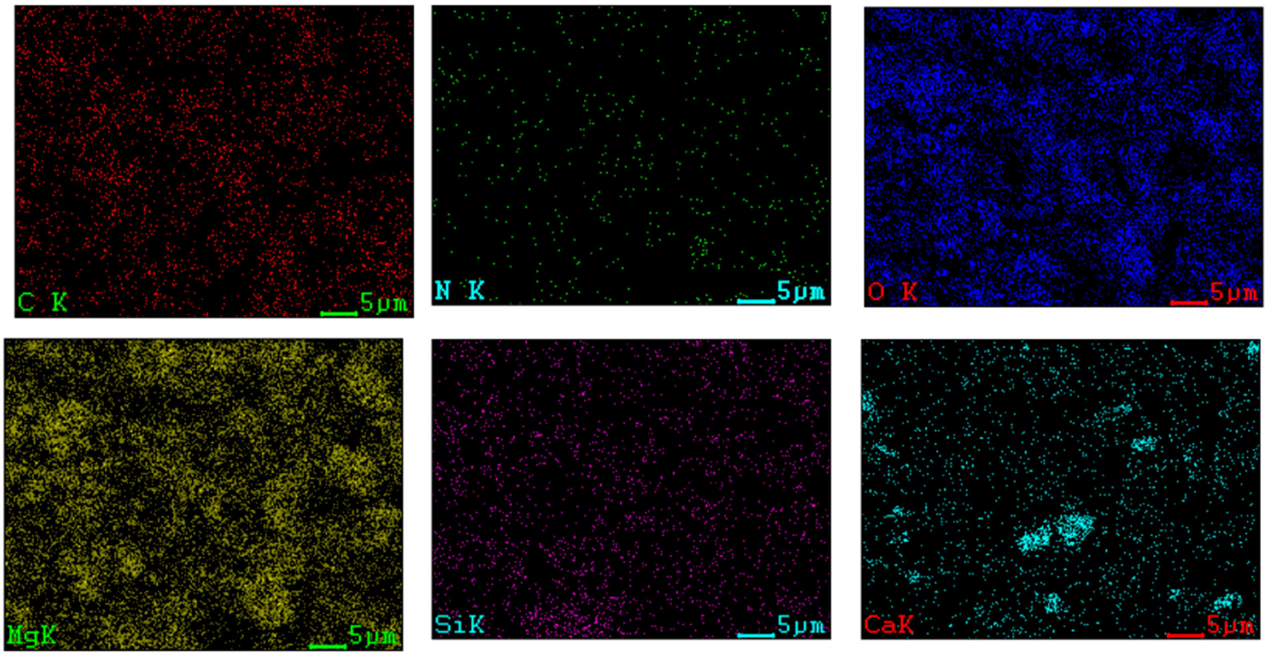

Figure S1: Element mapping by SEM/EDX of Tetra-Alg.

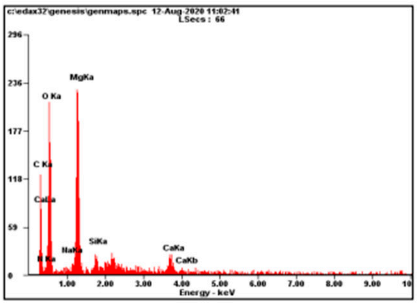

Element	Wt%	At%
CK	21.78	29.89
NK	01.90	02.23
OK	48.38	49.85
NaK	00.55	00.40
MgK	22.59	15.32
SiK	01.91	01.12
CaK	02.88	01.19
Matrix	Correction	ZAF

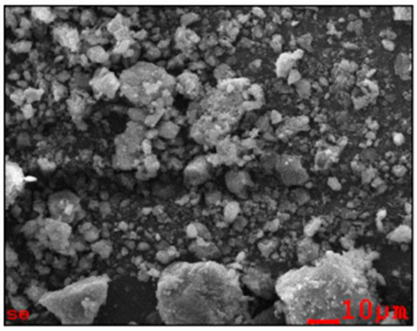

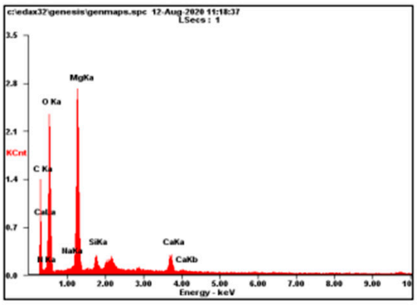

Element	Wt%	At%
CK	24.42	33.34
NK	01.07	01.25
OK	46.13	47.28
NaK	01.05	00.75
MgK	21.87	14.75
SiK	02.22	01.30
CaK	03.24	01.32
Matrix	Correction	ZAF

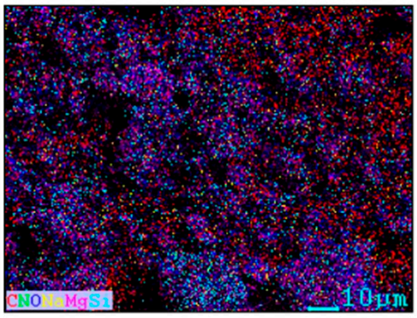

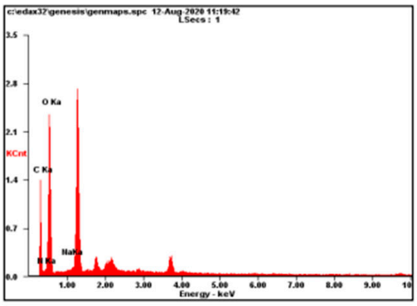

Element	Wt%	At%
CK	31.47	37.81
NK	06.54	06.74
OK	60.35	54.42
NaK	01.64	01.03
Matrix	Correction	ZAF

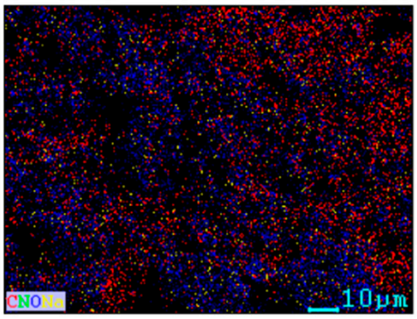

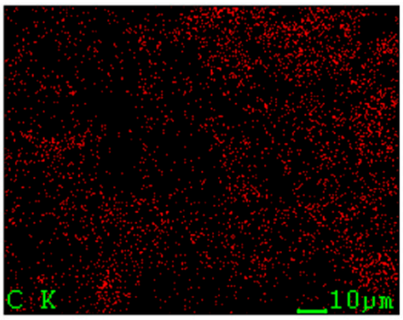

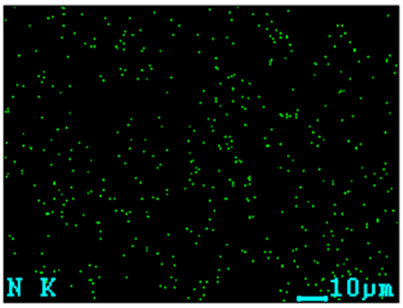

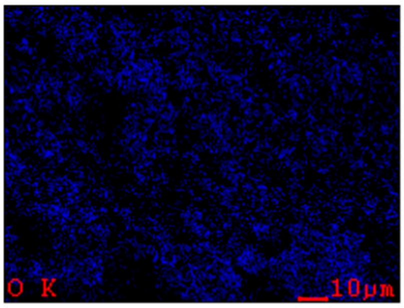

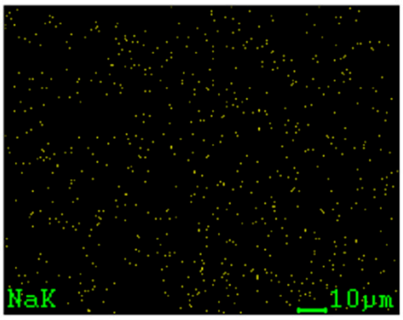

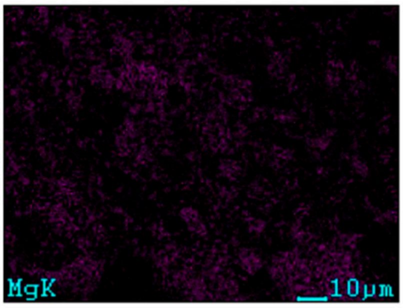

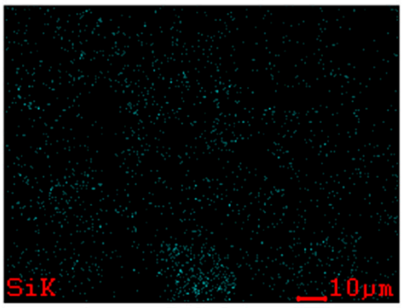

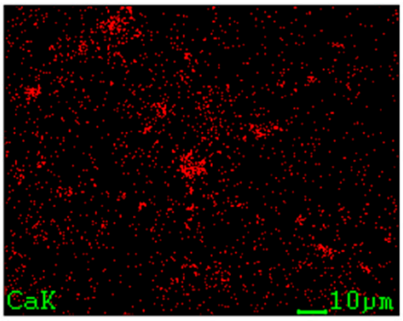

Figure S2: Element mapping by SEM/EDX of Tetra-Alg-Na

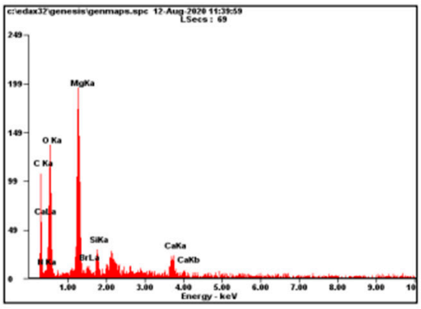

Element	Wt%	At%
CK	28.86	39.22
NK	00.00	00.00
OK	42.38	43.24
MgK	21.69	14.56
BrL	01.47	00.30
SiK	02.29	01.33
CaK	03.31	01.35
Matrix	Correction	ZAF

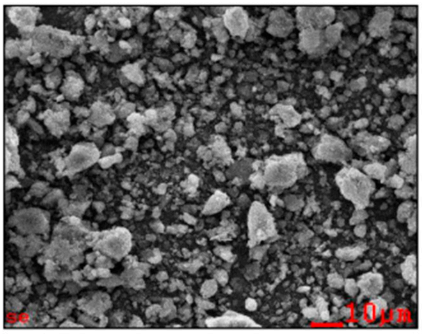

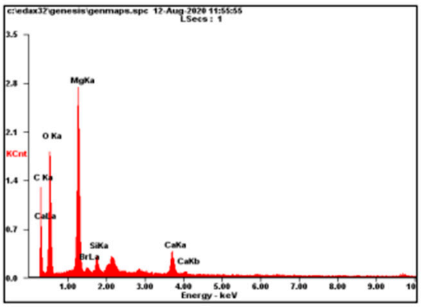

Element	Wt%	At%
CK	25.63	35.90
OK	42.81	45.01
MgK	22.05	15.26
BrL	02.95	00.62
SiK	02.58	01.54
CaK	03.99	01.68
Matrix	Correction	ZAF

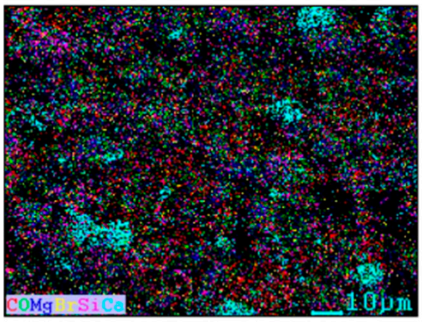

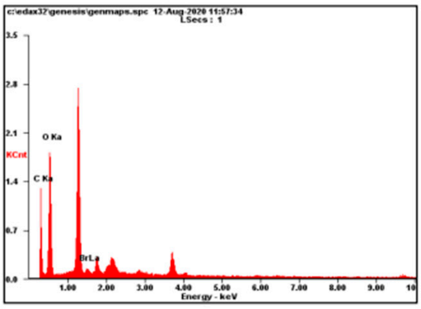

Element	Wt%	At%
CK	38.79	46.86
OK	57.94	52.55
BrL	03.26	00.59
Matrix	Correction	ZAF

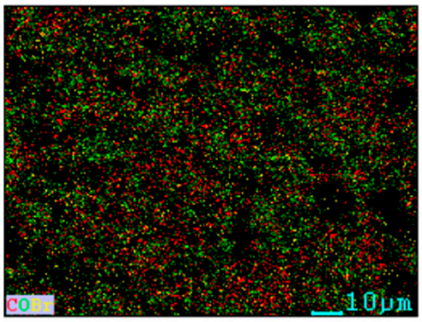

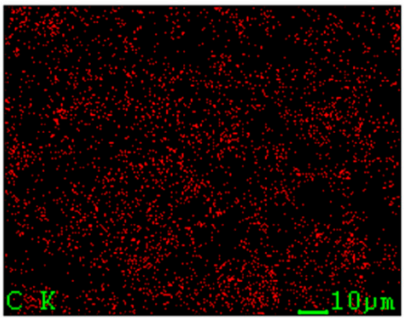

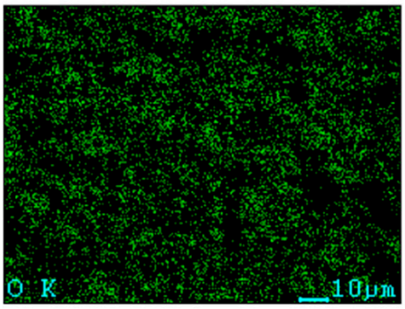

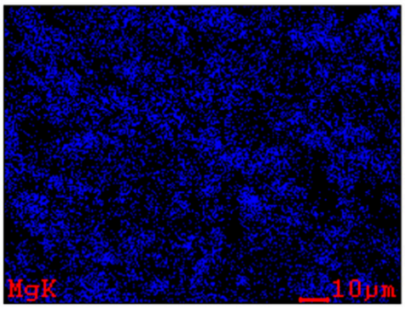

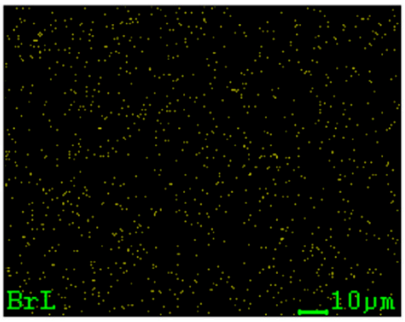

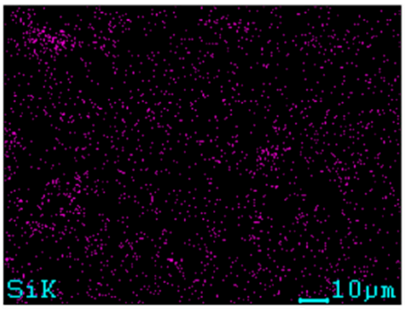

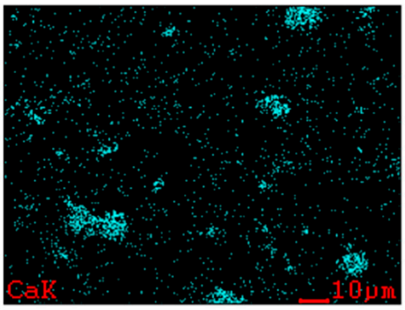

Figure S3: Element mapping by SEM/EDX of Tetra-Alg-CTAB.
